# Prevalence of Microorganisms of Public Health Significance in Ready-to-Eat Foods Sold in Developing Countries: Systematic Review and Meta-Analysis

**DOI:** 10.1155/2020/8867250

**Published:** 2020-11-01

**Authors:** Dechasa Adare Mengistu, Sina Temesgen Tolera

**Affiliations:** Department of Environmental Health, College of Health and Medical Science, Haramaya University, Harar, PO. Box 235 Dire Dawa, Ethiopia

## Abstract

**Background:**

The issue of microbial quality and safety of ready-to-eat foods has become a public health concern that needs to be addressed to protect the consumer's health. Contamination of ready-to-eat foods by enteric pathogens such as *Escherichia coli*, *Salmonella*, and *Staphylococcus aureus* bacteria is associated with potential health risks and can cause foodborne outbreaks. Thus, the systematic review and meta-analysis aimed at determining the overall evidence on the prevalence of microorganisms of public health significance in ready-to-eat foods based on previous studies.

**Methods:**

The articles published from 2015 to 2020 were searched from multiple electronic databases such as PubMed, Google Scholar, MEDLINE, CINAHL, Science Direct, Web of Science, and the Directory of Open Access Journals. The JBI critical appraisal tool was applied to the included articles. To determine the heterogeneity among the included articles, *I*^2^ statistics were used while publication bias was evaluated using the visual funnel plot. A Forest plot using the random effect model for meta-analysis was used to estimate the pooled prevalence of *E. coli*, *Salmonella*, and *S. aureus* in ready to eat foods.

**Results:**

The pooled prevalence of *E. coli*, *Salmonella*, and *S. aureus* in ready to eat foods was 33.8% (95% CI: 19.9, 51.2; *Q* value = 67.080, *I*^2^ = 89.56%), 26.0% (95% CI: 13.8, 43.6%; *Q* value = 83.67, *I*^2^ = 91.63%), and 46.3% (95% CI: 24.8, 69.4%, *I*^2^ = 94.9%), respectively.

**Conclusion:**

The findings show that contamination of ready-to-eat foods with pathogenic microorganisms continues to be a public health risk. Thus, effective food hygiene and safety systems are necessary to protect the health of the consumers and the public as a whole.

## 1. Background

Ready-to-eat foods (RTF) are defined as foods being ready for consumption that could be raw or cooked, hot, or chilled and can be consumed without further treatment or any processing [1, 2]. The consumption of various types of ready-to-eat foods in public places has become common worldwide. Due to the vital role of foods in human existence, it is important to maintain food safety to ensure that human being is safe from foodborne diseases or other related health hazards [3].

Ready-to-eat foods play a vital role in meeting the food requirements of many inhabitants and being appreciated by consumers for their affordability, accessibility, variety, and unique organoleptic properties [4–6]. However, unless they are handled under hygienic conditions and safety, ready-to-eat foods can serve as a good medium for the growth and multiplication of various pathogenic microorganisms of public health concern. The World Health Organization estimated more than 200 various types of diseases or illnesses caused or spread by foods [7]. However, the most common foodborne pathogen includes but is not limited to *Bacillus cereus*, *Clostridium botulinum*, *Escherichia coli*, *Shigella* spp., *Salmonella spp.*, *Staphylococcus aureus*, and *Campylobacter* [8].

Currently, the incidence of foodborne illness involving a broad range of diseases caused by pathogenic microorganisms is rising worldwide and becomes a public health concern [9, 10]. And foodborne illnesses are an important challenge to public health and cause a significant economic problem in many countries [11]. In developed countries, an estimated one-third of the population is affected by foodborne diseases each year. However, foodborne diseases are common and leading causes of illness in developing countries because of the prevailing poor hygienic and sanitation conditions or practices, weak food safety and regulatory systems, lack of resources, and lack of education [12, 13]. In response to these problems, health and other concerned organizations are increasing their effort to improve the quality and safety of foods and to prevent foodborne disease [14]. Thus, it is essential to provide the overall evidence on the prevalence of microorganisms of public health concern in ready-to-eat foods.

## 2. Objective

The study aimed to systematically review and provide the overall current evidence on the prevalence of microorganisms of public health concerns in ready-to-eat foods in developing countries.

## 3. Method

This systematic review and meta-analysis was conducted under the Preferred Reporting Items for Systematic Reviews and Meta-Analysis (PRISMA) guidelines [15].

### 3.1. Eligibility Criteria

The articles that met the following inclusion criteria were included in the systematic review and meta-analysis:

- *Study Area*. Research articles conducted in developing countries.
- *Study Design*. Cross-sectional studies reported the prevalence of microorganisms of public health concern in ready-to-eat foods such as *Salmonella*, *S. aureus*, and *E. coli*.
- *Language*. Full-text articles published in the English language.
- *Population*. Articles conducted on any type of ready-to-eat foods in developing countries.
- *Publication Issue*. Primary research articles published in peer-reviewed journals from 2015 to 2020.

### 3.2. Outcome Measure

The outcome of this systematic review and meta-analysis is to determine the pooled prevalence of *Escherichia coli*, *Salmonella*, and *Staphylococcus aureus* in ready-to-eat foods in developing countries. The prevalence of selected microorganisms was calculated by dividing the number of positive ready-to-eat foods samples to the total number of ready-to-eat foods samples investigated or analyzed (total sample size) multiplied by 100.

### 3.3. Information Sources and Search Strategy

The articles published from 2015 to 2020 were identified through a literature search of electronic databases such as Google Scholar, PubMed, MEDLINE, CINAHL, Science Direct, Web of Science, and the Directory of Open Access Journals. From PubMed databases, articles were searched using a combination of Boolean logic operators (AND, OR, and NOT), Medical Subject Headings (MeSH), and keywords. The searches were done using the keywords and Boolean logic operators as following: (Prevalence^∗^ OR Occurrence^∗^ OR frequency∗ OR Contamination) AND (Microorganisms∗OR Bacterial^∗^ OR Microbial^∗^ OR foodborne pathogens) AND (Public^∗^ OR Health^∗^ OR Public health) AND (significance∗ OR concern^∗^ OR hazards^∗^ OR risk) AND (ready to eat^∗^ OR street ^∗^ OR fast^∗^ OR cooked^∗^ OR processed^∗^ OR prepared^∗^ OR packed) AND (foods^∗^ OR meal^∗^ OR meat^∗^ OR fruit^∗^ OR fruit products ^∗^OR dairy product^∗^ OR vegetable ^∗^OR vegetable products, etc..) AND (Sub-Saharan ^∗^OR Developing^∗^ OR low income ^∗^OR middle income) AND (Countries^∗^ OR ^∗^ OR region, Africa, etc.).

The identified keywords and index terms were checked by authors (Mengistu DA and Tolera ST) across the included electronic databases. Additionally, manual searching for further studies was done by authors (Mengistu DA and Tolera ST) to cover other published articles. The last search was done on April 20, 2020.

### 3.4. Study Selection

Duplicated studies were removed using the ENDNOTE software version X5 (Thomson Reuters, USA). The authors (Mengistu DA and Tolera ST) individually screened all identified articles based on their titles and abstracts by applying the eligibility criteria. Disagreements were solved by taking the mean score of the two reviewers (Mengistu DA and Tolera ST) after discussing the rationale on differences and repeating the review procedure.

### 3.5. Data Extraction

All required and relevant data were extracted from the included articles using a predetermined data abstraction form by authors (Mengistu DA and Tolera ST) independently. The extracted data include the name of the authors, sample size, the primary outcome (prevalence of selected microorganisms of public health significance), countries where the article was carried out, year of publication, and study design.

### 3.6. Quality Assessment

For articles met inclusion criteria, abstracts were checked to establish their relevance for the study. The quality of the included articles was assessed using independent appraisal tools (JBI Critical Appraisal tools) [16]. Then, the score was taken across all the studies and graded as high (75% and above score), moderate (50-75% score), and low (<50% score) quality. High quality in this work indicates a low risk of bias. Disagreements made among authors (Mengistu DA and Tolera ST) on what is to be extracted were solved by discussion. Finally, the authors found all articles with a low risk of bias.

### 3.7. Statistical Procedure

The required data were extracted using a Microsoft Excel format, 2016. After the extraction, the data were imported to the Comprehensive Meta-Analysis (CMA) V3 statistical package (software). Then, the characteristics of the original articles were described using texts, tables, and forest plots. Heterogeneity among the reported prevalence was checked by using a heterogeneity *I*^2^ test.

The random-effect model of meta-analysis and forest plot was used to estimate the pooled prevalence of microorganisms of public health concern in ready-to-eat foods with 95% confidence intervals. The possibility of publication bias was assessed by visual funnel plots, and a *p* value of < 0.05 was considered as the evidence for publication bias. Moreover, subgroup analysis was done based on the countries where the included articles were conducted and publication year to minimize the random variations (heterogeneity) between the point estimates of the included articles.

## 4. Result

### 4.1. Study Selection

**Figure 1:**
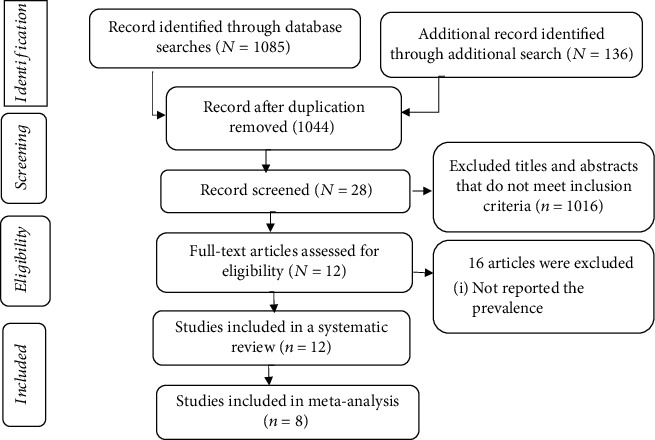
PRISMA flow diagram indicating the selection process of included articles for a systematic review and meta-analysis, 2020.

A total of 1221 articles published from 2012 to 2020 were identified using electronic databases and hand searching. After an initial screening of articles by their titles and abstracts, 149 duplicate articles were excluded, while 1044 studies were excluded based on the predetermined inclusion and exclusion criteria. Then, the full texts of the remaining 28 articles were further assessed to determine their eligibility for the systematic review and meta-analysis. Additionally, 16 articles were excluded as they failed to report the prevalence of selected microorganisms of public health concern. Twelve original articles that meet the predetermined inclusion criteria were included in the systematic review, of which 8 articles were included in quantitative analysis (meta-analysis) (Figure 1).

### 4.2. Study Characteristics

In this study, a total of 625 ready-to-eat food samples were included in eight articles published from 2015 to 2020 that were conducted in seven different developing countries: one in Ethiopia, two in Nigeria, one in India, one in Pakistan, one in Sudan, one in Namibia, and one in South Africa. All the included articles were cross-sectional studies with a sample size ranging from 15 to 205 ready-to-eat foods. In addition, based on JBI Critical Appraisal tool [16], all the included articles had a low risk of bias (Table 1).

The prevalence of *E. coli*, *Salmonella*, and *S. aureus* in ready-to-eat foods was in the range of 1.8% (24) to 100% (21), 2.0% (19) to 73.3% (21), and 7.0% (22) to 100% (21), respectively (Table 1). Overall, about 172 (27.5%), 168 (26.9%), and 259 (41.4%) of ready-to-eat food samples investigated in eight articles were contaminated with *Salmonella*, *E. coli*, and *S. aureus*, respectively.

### 4.3. Data Synthesis and Statistical Analysis

#### 4.3.1. Prevalence of Microorganisms of Public Health Concerns in Ready-to-Eat Foods

We conducted a meta-analysis using the Comprehensive Meta-Analysis (CMA) V3 statistical package to determine the pooled prevalence of microorganisms of public health concern in ready-to-eat foods.

**Figure 2:**
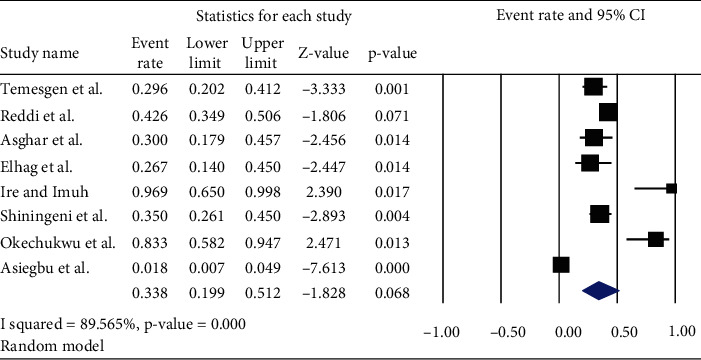
Forest plot shows the pooled prevalence of *E. coli* in ready-to-eat foods in developing countries, 2020.

*(1) Prevalence of E. Coli*. The pooled prevalence of *Escherichia coli* in ready-to-eat foods of developing countries was found to be 33.8% (95% CI: 19.9, 51.2; *Q* value = 67.080, *I*^2^ = 89.56%) (Figure 2).

Based on a subgroup analysis of included articles by country, the lowest prevalence [1.8% (95% CI: 0.7%, 4.9%) with a *p* value of < 0.001] of *E. coli* in ready-to-eat foods was observed in South Africa whereas the highest prevalence [88.8%, (95% CI: 62.5-97.40%) with a *p* value of 0.009] of *E.coli* in ready-to-eat foods was observed among the studies conducted in Nigeria.

**Figure 3:**
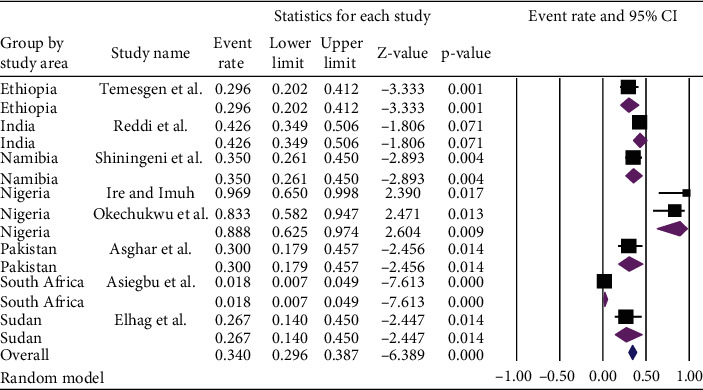
Subgroup analysis of the pooled prevalence of *E. coli* in ready-to-eat foods based on countries of developing countries, 2020.

However, after subgroup analysis by country, the overall pooled prevalence of *E. coli* in ready-to-eat foods was [34.0% (95% CI: 29.6-38.7%) with a *p* value of < 0.001] (Figure 3).

**Figure 4:**
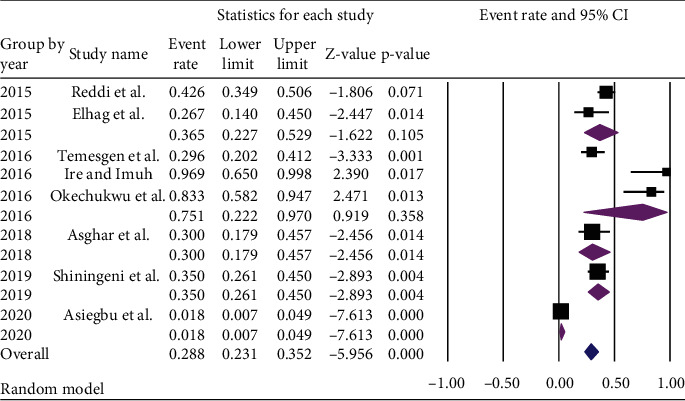
Subgroup analysis of the pooled prevalence of *E. coli* in ready-to-eat foods in developing countries by the year of publication, 2020.

Regarding the publication year, the pooled prevalence of *E. coli* was higher [75.1% (95% CI: 22.2-97.0%)] among the studies published in 2016, while the article published in 2020 reported lower [1.8% (95% CI: 0.7%, 4.9%) with *p* value of < 0.001] prevalence (Figure 4).

**Figure 5:**
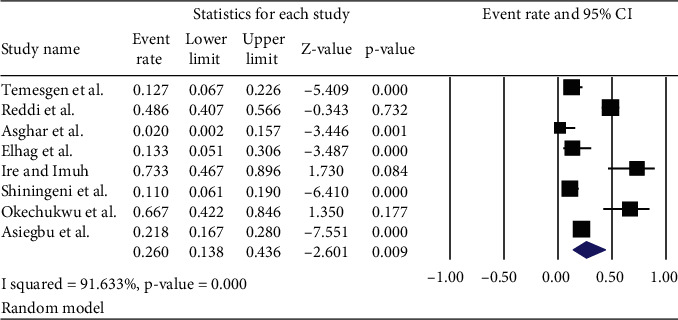
The pooled prevalence of *Salmonella* in ready-to-eat foods in developing countries, 2020.

*(2) Prevalence of Salmonella*. The pooled prevalence of *Salmonella* in ready-to-eat foods of developing countries was found to be 26.0% (95% CI: 13.8, 43.6%; *Q* value = 83.67, *I*^2^ = 91.63%) (Figure 5).

**Figure 6:**
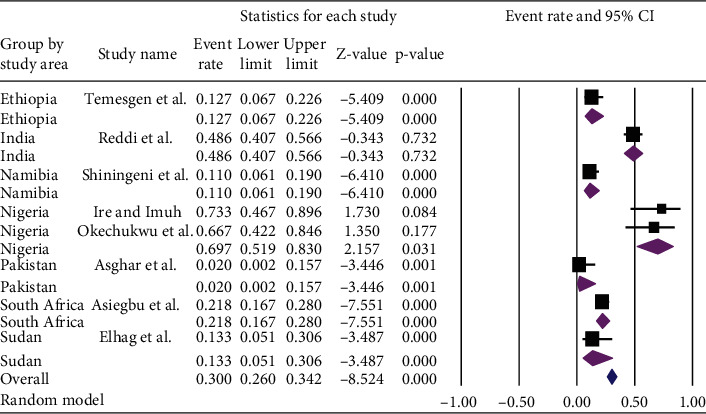
Subgroup analysis of the pooled prevalence of *Salmonella* in ready-to-eat foods in developing countries by countries, 2020.

Among all countries, the lowest prevalence [2.0% (95% CI: 0.2%, 15.7%) with a *p* value of = 0.001] of *Salmonella* in ready-to-eat foods was observed in Pakistan whereas the highest prevalence [69.7%, (95% CI: 51.9-83.0%) with a *p* value of 0.031] of *Salmonella* in ready-to-eat foods was observed in Nigeria. After subgroup analysis, the overall pooled prevalence of *Salmonella* was found to be [30.0% (95% CI: 26.0-34.2%) with *p* value of < 0.001] (Figure 6).

**Figure 7:**
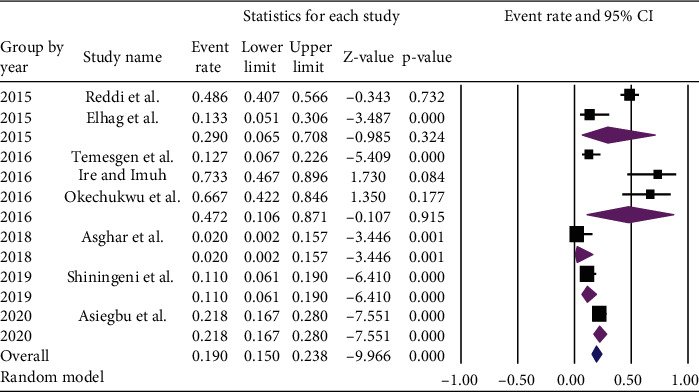
Subgroup analysis of the pooled prevalence of *Salmonella* in ready-to-eat foods in developing countries by the year of publication, 2020.

Subgroup analysis based on publication year and prevalence of *Salmonella* among ready-to-eat foods was done. The higher pooled prevalence of *Salmonella* [47.2% (95% CI: 10.6-87.1%)] was found among those articles published in 2016, whereas the article conducted in 2018 reported lower [2.0% (95% CI: 0.2%, 15.7%) with *p* value of = 0.001] prevalence (Figure 7).

**Figure 8:**
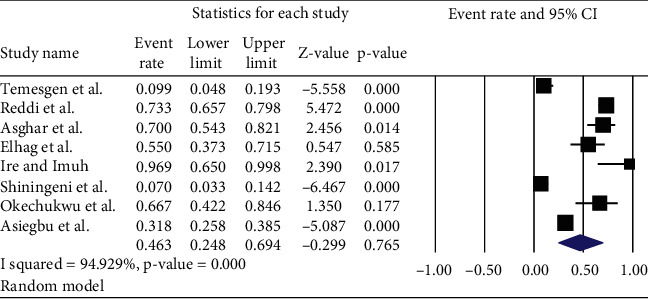
The pooled prevalence of *Staphylococcus aureus* in ready-to-eat foods in developing countries, 2020.

*(3) Prevalence of Staphylococcus Aureus*. The pooled prevalence of *Staphylococcus aureus* in ready-to-eat foods of developing countries was found to be 46.3% (95% CI: 24.8, 69.4%, *I*^2^ = 94.9%) (Figure 8).

**Figure 9:**
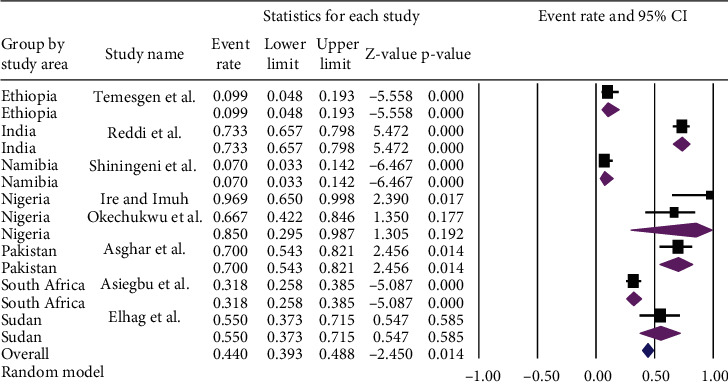
Subgroup analysis of the pooled prevalence of *Staphylococcus aureus* in ready-to-eat foods in developing countries by countries, 2020.

A subgroup analysis was performed based on the countries where the articles were conducted and the year of publication. Among the included countries, the lowest prevalence [7% (95% CI: 3.3%, 14.2%) with a *p* value of < 0.001] of *Staphylococcus aureus* in ready-to-eat foods was observed in Namibia whereas the highest prevalence [85%, (95% CI: 29.5-98.7%)] of *Staphylococcus aureus* in ready-to-eat foods was observed in Nigeria. However, after subgroup analysis, the overall prevalence of *Staphylococcus aureus* was found to be [44.0% (95% CI: 39.3, to 48.8%) with a *p* value of < 0.014] prevalence (Figure 9).

**Figure 10:**
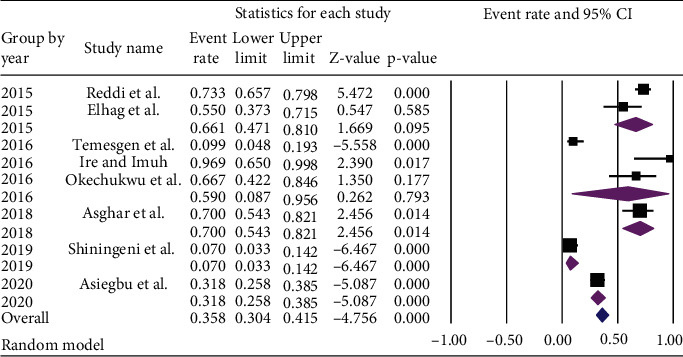
Subgroup analysis of the pooled prevalence of *Staphylococcus aureus* in ready-to-eat foods in developing countries by the year of publication, 2020.

Subgroup analysis based on the publication year of included articles found the lower pooled prevalence of *Staphylococcus aureus* [7.0% (95% CI: 3.3%, 14.2%) with *p* value of < 0.001] among studies published in 2019, while the article published in 2018 reported higher prevalence [70.0% (95% CI: 54.3%, 82.1%) with *p* value of = 0.014] (Figure 10).

## 5. Discussion

Various studies conducted across the world are strongly agreeing with the fact that most of the pathogenic microorganisms of public health concerns are introduced to foods during handling, processing, and preparation [25]. These microorganisms have potential health risks to consumers, particularly in developing countries, and need to be addressed to protect the health and wellbeing of the public.

**Figure 11:**
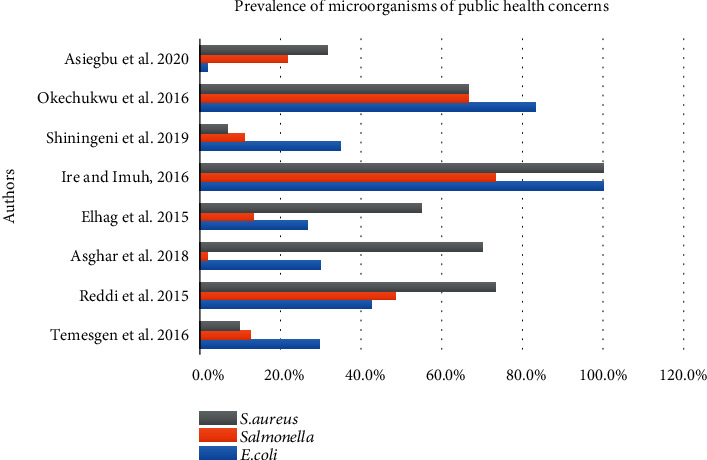
Show the reported prevalence of *E. coli*, *Salmonella*, and *Staphylococcus* in ready-to-eat foods in developing countries.

Among 8 articles included in this review, Ire and Imuh 2016 reported a higher prevalence of *E. coli* and *S. aureus* than other included articles, while Asiegbu et al., 2020, reported lower *E. coli*. However, the highest and lowest prevalence of *Salmonella* was reported by Ire and Imuh, 2016, and Asghar et al., 2018, respectively (Figure 11).

The difference in microorganisms may be related to poor hygiene and safety practices or due to contamination of raw materials used or water supply used or the lifestyle of the community where the study was conducted.

The current review found the overall prevalence of positive ready-to-eat foods in terms of *E. coli*, *Salmonella*, and *S. aureus* that accounts for 172 (27.5%), 168 (26.9%), and 259 (41.4%), respectively. However, quantitative analysis of the included articles indicated the pooled prevalence of *Escherichia coli* in ready-to-eat foods accounts 33.8%, while the pooled prevalence of *Salmonella* and *Staphylococcus* accounts for 26.0% and 46.3%, respectively. Another quantitative analysis (meta-analysis) conducted in selected African countries was agreed with the current finding in terms of pooled prevalence of *E. coli* (31.6%). However, found lower pooled prevalence of *Salmonella* (21.7%) and Staphylococcus aureus (25.1%) among ready to eat foods than the current finding [26]. The variation may be due to poor hygiene, safety and sanitation conditions, low quality of raw materials used, lack or inadequate training on food hygiene, and safety for food handlers.

## 6. Conclusion

This systematic review and meta-analysis estimated the pooled prevalence of *E. coli*, *Salmonella*, and *Staphylococcus aureus* in ready-to-eat foods in developing countries. The findings show that contamination of ready-to-eat foods with pathogenic microorganisms continues to be a public health risk that needs to be addressed to protect the health and wellbeing of the consumers and the public as a whole. Thus, effective food hygiene and safety systems and tailor training both at the national and international level are necessary to prevent foodborne illness/disease and to protect the health of the consumers and the public as a whole.

## Figures and Tables

**Table 1:** Overall characteristics of included articles in systematic review and meta-analysis, 2020.

Authors	Publication Year	*N*	Study design	Prevalence of selected microorganisms			Country	Risk of bias	References
				*E. coli*	*Salmonella*	*S. aureus*			
Temesgen et al.	2016	71	Cross-sectional	29.6%	12.7%	9.9%	Ethiopia	Low	[17]
Reddi et al.	2015	150	Cross-sectional	42.6%	48.6%	73.3%	India	Low	[18]
Asghar et al.	2018	40	Cross-sectional	30%	2.0%	70.0%	Pakistan	Low	[19]
Elhag et al.	2015	30	Cross-sectional	26.7%	13.3%	55%	Sudan	Low	[20]
Ire and Imuh	2016	15	Cross-sectional	100%	73.3%	100%	Nigeria	Low	[21]
Shiningeni et al.	2019	96	Cross-sectional	35.0%	11.0%	7.0%	Namibia	Low	[22]
Okechukwu et al.	2016	17	Cross-sectional	83.3%	66.7%	66.7%	Nigeria	Low	[23]
Asiegbu et al.	2020	205	Cross-sectional	1.8%	21.8%	31.8%	South Africa	Low	[24]

*N*: sample size; *E. coli*: *Escherichia Coli*; *S. aureus*: *Staphylococcus aureus.*

## Data Availability

All data are included in the systematic review and meta-analysis. In addition, the Supplementary materials including PRISMA-P (Preferred Reporting Items for Systematic Review and Meta-Analysis Protocols) 2015 checklist are the recommended items to address in a systematic review protocol.

## Abbreviations

CMA: Comprehensive Meta-Analysis
JBI: Joanna Briggs Institute
PRISMA: Preferred Reporting Items for Systematic Review and Meta-Analysis
RTE: Ready to eat
WHO: World Health Organization.

## Supplementary Materials

Supplementary Materials PRISMA-P (Preferred Reporting Items for Systematic Review and Meta-Analysis) 2015 checklist is one of the recommended items to address in a systematic review.
